# Stress hyperglycemia ratio and neutrophil to lymphocyte ratio are reliable predictors of new-onset atrial fibrillation in patients with acute myocardial infarction

**DOI:** 10.3389/fcvm.2022.1051078

**Published:** 2022-11-09

**Authors:** Lifei Pan, Zhitong Li, Chenglin Li, Xiaopeng Dong, Tesfaldet H. Hidru, Fei Liu, Yunlong Xia, Xiaolei Yang, Lei Zhong, Ying Liu

**Affiliations:** Department of Cardiology, First Affiliated Hospital of Dalian Medical University, Dalian, China

**Keywords:** stress hyperglycemia ratio, neutrophil to lymphocyte ratio, acute myocardial infarction, atrial fibrillation, diabetes mellitus

## Abstract

**Background:**

The occurrence of new-onset atrial fibrillation (NOAF) post-acute myocardial infarction (AMI) is associated with worse outcomes. In this study, we sought to assess the predictive effect of stress hyperglycemia ratio (SHR) and neutrophil to lymphocyte ratio (NLR) to predict NOAF in patients with AMI.

**Materials and methods:**

We recruited 3,194 individuals with AMI but free of atrial fibrillation (AF). AMI cases were stratified into groups according to SHR and NLR quartiles and were further categorized based on diabetes status. High SHR and high NLR were defined as the highest quartile of SHR and NLR. A nomogram incorporating risk factors for NOAF was constructed using multivariate logistic regression analyses. The performance of the novel nomogram was tested for predictive performance, agreement between the actual and predicted probability, and clinical utility using area under the curve (AUC), bootstrapped calibration curves, and decision curve analysis, respectively.

**Result:**

A total of 245 (7.67%) patients developed NOAF post-AMI. The NOAF cases had higher values of SHR and NLR than non-NOAF patients after AMI regardless of diabetes status. After adjusting for potential confounders, high SHR and NLR were independently associated with NOAF post-AMI. Moreover, the novel nomogram incorporating high NLR and high SHR for NOAF risk estimation in patients with AMI showed satisfactory performance assessed by the AUC, calibration curves, decision curve analysis.

**Conclusion:**

SHR and NLR were independently associated with NOAF in AMI patients. The constructed novel nomogram that incorporates SHR and NLR might assist in NOAF risk stratification post-AMI.

## Introduction

Among many complications of acute myocardial infarction (AMI), new-onset atrial fibrillation (NOAF) is popular. The incidence of NOAF ranges from 6 to 21% and its presence predicts an increased risk of death (1, 2). However, the development of NOAF is complex and the mechanisms are still unclear (2, 3). Therefore, identifying high-NOAF-risk patients earlier could be helpful for better prognosis prediction, and tailoring the individual therapy strategy.

Stress hyperglycemia is regarded as the transient increase in blood glucose concentrations. It is suggested that stress hyperglycemia link with worse outcomes in cases of AMI regardless of diabetic status (4). In the past, many studies have utilized admission blood glucose (ABG) to mark stress hyperglycemia levels. However, ABG can be influenced by acute physiological stress or the presence of a chronic rise in baseline glucose levels (5). Consequently, Roberts et al. (6) proposed stress hyperglycemia ratio (SHR) to control bias related to background glycemia or acute hyperglycemia. In fact, SHR has been suggested as an accurate index to identify stress hyperglycemia than ABG and exhibited better predictive value in the prognosis of AMI (7). However, the predictive value of SHR on the risk of NOAF after AMI has been rarely reported.

Neutrophil to lymphocyte ratio (NLR), a surrogate marker of the state of systematic inflammation, has been widely studied. Established evidence indicates that NLR was closely linked to slow progress of cardiovascular events (CVE) or increased severity of CVD conditions (8, 9) and this association was more significant than neutrophil or lymphocyte alone and various leukocyte parameters (10). However, the predictive value of NLR for NOAF in patients with AMI has been less investigated.

As such, the current study is investigated to evaluate the independent predictive value of the SHR and NLR for NOAF in patients with AMI. Furthermore, in this study, we established a quantitative method for clinicians to predict the incidence of NOAF in patients with AMI by nomogram analysis.

## Materials and methods

### Study participants

**FIGURE 1:**
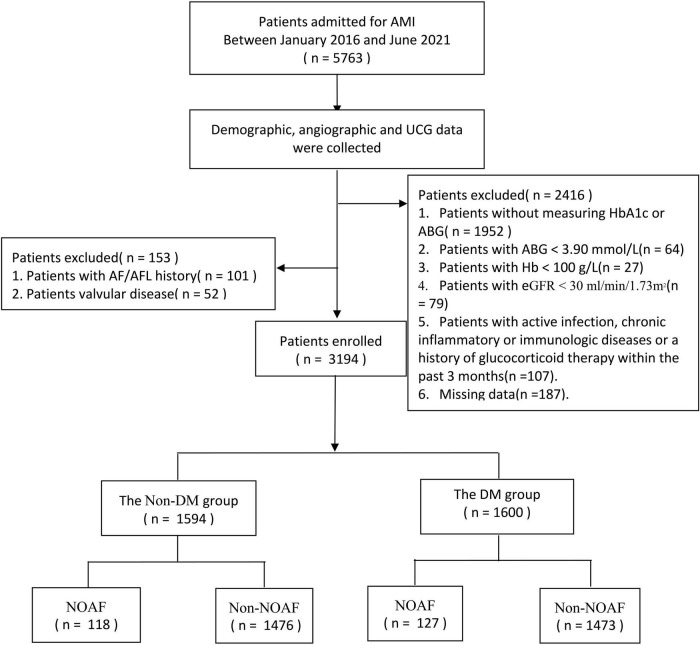
Flow chart. AMI, acute myocardial infarction; UCG, ultrasound cardiogram; AF, atrial fibrillation; AFL, atrial flutter; HbA1c, hemoglobin A1c; ABG, admission fasting blood gulcose; eGFR, estimated glomerular filtration rate; DM, diabetes mellitus; NOAF, new-onset atrial fibrillation.

The present study was a sub analysis of a previous study (11). The study was conducted based on clinical examinations carried out among AMI patients at the First Affiliated Hospital of Dalian Medical University (FAHDMU) between January 2016 and June 2021. We initially considered 5,763 AMI patients for coronary arteriography (CAG). We excluded participants with (i) a history of atrial fibrillation (AF) and/or atrial flutter (AFL) (*n* = 101), (ii) a history of cardiac valvular disease (*n* = 52), (iii) fasting blood glucose (FBG) < 3.9 mmol/L (*n* = 64), Hb < 100 g/L (*n* = 27), (iv) estimate glomerular filtration rate (eGFR) < 30 ml/min/1.73 m^2^ (*n* = 79), (v) active infection, chronic inflammatory, or immunologic disease or a history of glucocorticoid therapy within the past 3 months (*n* = 107), and (vi) lack of crucial laboratory data (glucose or HbA1c results) or other clinical data (*n* = 2,139). Finally, 3,194 patients were included in the analysis (Figure 1). The study was approved by the Ethics Committee of FAHDMU and complied with the principles of the Declaration of Helsinki. The electronic medical record of FAHDMU was utilized to retrieve data, therefore our study waived the obligation for informed consent.

### Definition

In our study, those AMI cases with history of diabetes or HbA1c value of >6.5% were considered to have diabetes mellitus (DM) (12). Hypertension (HTN) was defined based on the rise of blood pressure (SBP ≥ 140 mmHg or DBP ≥ 90 mmHg) and/or positive history of antihypertensive drug use (13). Congestive heart failure (CHF) was defined as per previous studies (14). Elevated cardiac troponin values accompanied by clinical evidence for myocardial ischemia, presence of pathological Q wave on electrocardiogram (ECG), or evidence of new regional wall motion abnormality in echocardiography was defined as acute myocardial infarction (15). The SHR was calculated as the admission FBG divided by the average glucose: SHR = admission FBG/[1.59 × HbA1c (%) - 2.59] (16). The NLR output was computed as plasma neutrophil count (*10^9^/L)/plasma lymphocyte count (*10^9^/L) (17). AF was diagnosed as the absence of P waves, coarse or fine fibrillatory waves, and irregular RR intervals (18). If there was any proof of AF from the electrocardiography readings during the hospitalization period in AMI cases with no previous history of AF/AFL, we referred to them as new-onset AF cases.

### Statistical analysis

The data for this study were analyzed using SPSS software (V.24.0, IBM, New York, NY, USA) and R software (V.4.0, R Foundation for Statistical Computing, Vienna, Austria). The population studied was stratified by diabetes status and the presence of NOAF. The distribution of continuous variables was evaluated by using the Kolmogorov–Smirnov test. The output of the normally distributed variables was presented as mean ± standard deviation (SD) whereas the output of the variables with skewed distribution was expressed as median (25th–75th interquartile). Categorical variables were expressed using frequency and percentage. Based on the results from Kolmogorov–Smirnov test, continuous data were compared using the Mann–Whitney U test or independent-samples *T*-test. For categorical variables, the chi-square test or Fisher exact test was used for comparison analysis and presented as a proportion. To compare the incidence of NOAF after AMI at different SHR and NLR levels, patients were further divided into four groups according to quartiles (Q) of SHR and NLR levels at baseline. High SHR and high NLR were defined as the highest quartile of SHR and NLR. We run binary logistic regression analysis to estimate the risk of NOAF for Q2, Q3, and Q4 of NLR and SHR, with Q1 of NLR/SHR serving as the reference for comparison. Only these variables with a *P*-value < 0.05 in the univariate analysis were included in the multivariate logistic model.

In this study, rms package of R, version 4.0^1^ was used to construct a nomogram. The area under the receiver operating characteristic curve AUC was used to assess the predictive capacity of the novel nomogram. We estimated the bootstrapped calibration curves (1,000 resampling) to reflect the consistency between the actual and predicted probability. The decision curve analysis was checked to understand whether the net advantage of the nomogram remains appropriate for clinical use. A two-tailed *P*-value < 0.05 was considered statistically significant.

## Results

### Baseline characteristics of the study participants

As shown in Table 1, 3,194 participants with AMI were enrolled in this research and stratified by diabetes status and the presence of NOAF. NOAF patients accounted for 7.67% (245/3,194) of the overall cohort, 7.94% (127/1,600) of DM patients, and 7.40% (118/1,594) of non-DM patients, respectively. Among these patients, 72 patients had AF/AFL rhythm at admission, the remaining patients were in sinus rhythm (SR). The baseline characteristics were comparable between participants with SR and AF/AFL rhythm at admission except for a higher heart rate, larger left atrium diameter, and longer hospitalization duration. At discharge, participants with AF/AFL rhythm at admission received more oral anticoagulants prescription and less antiplatelet drug prescription than those with NOAF. However, we found no significant difference in SHR and NLR in the two groups (Supplementary Table 1).

**TABLE 1:** Baseline characteristics.

	Non-DM				DM			
Variable	Total (*n* = 1,594)	SR (*n* = 1,476)	NOAF (*n* = 118)	*P-value*	Total (*n* = 1,600)	SR (*n* = 1,473)	NOAF (*n* = 127)	*P-value*
Age, years	62.0 (12.4)	61.4 (12.3)	69.4 (10.8)	<0.001	64.0 (11.3)	63.5 (11.3)	69.8 (9.0)	<0.001
Male, *n* (%)	1,311 (82.2)	1,224 (82.9)	87 (73.7)	0.012	1,129 (70.6)	1,056 (71.7)	73 (57.5)	0.001
Smoking, *n* (%)	795 (49.9)	749 (50.7)	46 (39.0)	0.014	651 (40.7)	615 (41.8)	36 (28.3)	0.003
Drinking, *n* (%)	391 (24.5)	362 (24.5)	29 (24.6)	0.990	296 (18.5)	282 (19.1)	14 (11.0)	0.024
**Medical history**								
HTN, *n* (%)	846 (53.1)	783 (53.0)	63 (53.4)	0.943	1,057 (66.1)	962 (65.3)	95 (74.8)	0.030
Prior MI, *n* (%)	10 (0.6)	8 (0.5)	2 (1.7)	0.166	20 (1.3)	15 (1.0)	5 (3.9)	0.015
Prior PCI, *n* (%)	122 (7.7)	111 (7.5)	11 (9.3)	0.479	152 (9.5)	139 (9.4)	13 (10.2)	0.768
Previous stroke, *n* (%)	100 (6.3)	86 (5.8)	14 (11.9)	0.009	137 (8.6)	124 (8.4)	13 (10.2)	0.482
PAD, *n* (%)	135 (8.5)	121 (8.2)	14 (11.9)	0.169	180 (11.3)	163 (11.1)	17 (13.4)	0.427
CHF, *n* (%)	70 (4.4)	55 (3.7)	15 (12.7)	<0.001	179 (11.2)	154 (10.5)	25 (19.7)	0.002
**Initial presentation**								
OHCA, *n* (%)	25 (1.6)	20 (1.4)	5 (4.2)	0.041	17 (1.1)	15 (1.0)	2 (1.6)	0.892
SBP, mmHg	130.2 (23.7)	130.5 (23.4)	126.5 (26.8)	0.078	135.2 (23.6)	135.1 (23.5)	136.7 (23.8)	0.446
DBP, mmHg	78.6 (13.4)	78.8 (13.3)	75.8 (14.5)	0.017	79.3 (12.7)	79.1 (12.6)	81.8 (14.0)	0.023
HR at admission, bpm	74.2 (15.6)	73.3 (14.1)	84.8 (25.9)	<0.001	77.4 (15.0)	76.8 (14.3)	84.1 (20.9)	<0.001
KILLIP > 1, *n* (%)	220 (13.8)	182 (12.3)	38 (32.2)	<0.001	309 (19.3)	264 (17.9)	45 (35.4)	<0.001
STEMI, *n* (%)	806 (50.6)	737 (49.9)	69 (58.5)	0.074	726 (45.4)	669 (45.4)	57 (44.9)	0.907
Anterior wall, *n* (%)	303 (37.6)	277 (37.6)	26 (37.7)	0.987	265 (36.5)	242 (36.2)	23 (40.4)	0.529
Inferior wall, *n* (%)	276 (34.2)	253 (34.3)	23 (33.3)	0.868	256 (35.3)	235 (35.1)	21 (36.8)	0.795
Others, *n* (%)	125 (15.5)	113 (15.3)	12 (17.4)	0.651	105 (14.5)	98 (14.6)	7 (12.3)	0.626
CHA_2_DS_2_-VASc score	1 (1–2)	1 (1–2)	2 (1–3)	<0.001	3 (2–4)	3 (2–4)	4 (2–5)	<0.001
GRACE score	132.1 (34.1)	130.1 (33.1)	157.5 (35.2)	<0.001	133.6 (30.8)	132.2 (30.3)	149.3 (32.1)	<0.001
**Culprit lesion**								
LM, *n* (%)	35 (2.2)	31 (2.1)	4 (3.4)	0.553	35 (2.2)	31 (2.1)	4 (3.1)	0.648
LAD, *n* (%)	666 (41.8)	615 (41.7)	51 (43.2)	0.742	690 (43.1)	630 (42.8)	60 (47.2)	0.329
LCX, *n* (%)	288 (18.1)	268 (18.2)	20 (16.9)	0.743	306 (19.1)	282 (19.1)	24 (18.9)	0.946
RCA, *n* (%)	544 (34.1)	508 (34.4)	36 (30.5)	0.389	576 (36.0)	535 (36.3)	41 (32.3)	0.363
**Laboratory data**								
eGFR, ml/(min •1.73 m^2^)	90.5 (23.2)	91.2 (23.0)	82.5 (23.5)	<0.001	93.6 (28.3)	94.4 (28.3)	83.6 (26.4)	<0.001
BNP, pg/mL	115.3 (48.0–263.0)	106.1 (45.8–235.3)	297.0 (142.0–548.9)	<0.001	141.1 (57.7–347.6)	134.2 (54.0–320.4)	315.1 (118.8–704.7)	<0.001
hsTnI, pg/mL	11.5 (1.8–58.4)	10.8 (1.8–55.4)	36.0 (3.9–108.1)	0.001	7.8 (1.1–46.7)	7.5 (1.1–46.5)	14.1 (1.2–55.4)	0.342
Glucose, mmol/L	5.2 (4.8–5.8)	5.2 (4.8–5.7)	5.5 (4.9–6.1)	<0.001	8.5 (6.9–11.0)	8.4 (6.9–10.8)	9.3 (6.9–12.0)	0.057
HbA1c (%)	5.7 (5.5–6.0)	5.7 (5.5–6.0)	5.7 (5.5–6.0)	0.447	8.1 (7.0–9.5)	8.1 (7.0–9.5)	8.0 (7.1–9.4)	0.829
SHR	0.8 (0.7–0.9)	0.8 (0.7–0.9)	0.9 (0.7–0.9)	0.001	0.8 (0.7–1.0)	0.8 (0.7–1.0)	0.9 (0.7–1.1)	0.005
WBC, *10^9/L	8.6 (6.8–10.5)	8.5 (6.8–10.5)	9.4 (7.5–11.5)	0.004	8.5 (7.0–10.7)	8.5 (6.9–10.6)	8.3 (7.1–10.9)	0.712
Neutrophil, *10^9/L	6.1 (4.5–8.1)	6.0 (4.4–8.0)	7.2 (5.1–9.3)	<0.001	6.0 (4.5–8.1)	6.0 (4.4–8.0)	6.2 (4.8–8.7)	0.199
Lymphocyte, *10^9/L	1.6 (1.2–2.1)	1.6 (1.2–2.1)	1.4 (1.1–1.8)	0.005	1.7 (1.2–2.2)	1.7 (1.3–2.2)	1.4 (1.0–1.9)	<0.001
NLR	3.7 (2.5–5.9)	3.6 (2.4–5.7)	4.9 (3.0–8.4)	<0.001	3.5 (2.4–5.6)	3.4 (2.4–5.5)	4.6 (2.7–7.0)	<0.001
Hemoglobin, g/L	141 (130–151)	141 (131–151)	136 (127–146)	0.005	138 (126–149)	138 (126–149)	136 (123–145)	0.016
Platelet, *10^9/L	208 (176–244)	209 (177–246)	197 (158–224)	<0.001	210 (177–250)	211 (177–250)	205 (170–246)	0.415
TC, mmol/L	4.8 (4.1–5.5)	4.8 (4.1–5.5)	4.6 (4.0–5.3)	0.052	4.8 (4.0–5.6)	4.8 (4.0–5.6)	4.6 (3.9–5.6)	0.330
TG, mmol/L	1.4 (1.1–2.0)	1.5 (1.1–2.0)	1.2 (0.9–1.6)	<0.001	1.7 (1.2–2.5)	1.7 (1.3–2.5)	1.5 (1.1–2.1)	0.001
HDL, mmol/L	1.0 (0.9–1.2)	1.0 (0.9–1.2)	1.1 (0.9–1.3)	0.002	1.0 (0.8–1.2)	1.0 (0.8–1.2)	1.0 (0.9–1.2)	0.140
LDL, mmol/L	2.7 (2.3–3.3)	2.8 (2.3–3.3)	2.6 (2.0–3.1)	0.012	2.7 (2.2–3.3)	2.8 (2.2–3.3)	2.6 (2.1–3.2)	0.370
**Echocardiographic parameters**								
LAD, mm	37.1 (4.1)	36.9 (3.7)	39.7 (6.8)	<0.001	37.7 (3.7)	37.5 (3.6)	39.8 (4.2)	<0.001
LVEF, %	52.8 (7.6)	53.1 (7.5)	49.8 (8.3)	<0.001	51.2 (9.0)	51.5 (8.9)	48.0 (10.0)	<0.001
**Initial treatment**								
PCI, *n* (%)	1,340 (84.1)	1,249 (84.6)	91 (77.1)	0.032	1,374 (85.9)	1,276 (86.6)	98 (77.2)	0.003
CABG, *n* (%)	9 (0.6)	9 (0.6)	0 (0.0)	1.000	11 (0.7)	9 (0.6)	2 (1.6)	0.215
Thrombolysis, *n* (%)	10 (0.6)	8 (0.5)	2 (1.7)	0.166	15 (0.9)	14 (1.0)	1 (0.8)	1.000
Length of hospitalization, day	6 (5–7)	6 (5–7)	7 (6–9)	<0.001	6 (5–7)	6 (5–7)	7 (6–9)	<0.001
In-hospital death, *n* (%)	12 (0.8)	10 (0.7)	2 (1.7)	0.221	12 (0.8)	10 (0.7)	2 (1.6)	0.262
**Medication at discharge**								
ACEI/ARB, *n* (%)	1,065 (66.8)	991 (67.1)	74 (62.7)	0.326	1,153 (72.1)	1,063 (72.2)	90 (70.9)	0.754
β blocker, *n* (%)	1,233 (77.4)	1,128 (76.4)	105 (89.0)	0.002	1,335 (83.4)	1,222 (83.0)	113 (89.0)	0.080
Statins, *n* (%)	1,591 (99.8)	1,473 (99.8)	118 (100.0)	1.000	1,593 (99.6)	1,466 (99.5)	127 (100.0)	1.000
OAC, *n* (%)	37 (2.3)	13 (0.9)	24 (20.3)	<0.001	35 (2.2)	12 (0.8)	23 (18.1)	<0.001
Aspirin, *n* (%)	1,557 (97.7)	1,453 (98.4)	104 (88.1)	<0.001	1,571 (98.2)	1,455 (98.8)	116 (91.3)	<0.001
P2Y_12_ receptor inhibitor, *n* (%)	1,587 (99.6)	1,472 (99.7)	115 (97.5)	0.011	1,598 (99.9)	1,473 (100.0)	125 (98.4)	0.006
Diuretic, *n* (%)	432 (27.1)	375 (25.4)	57 (48.3)	<0.001	551 (34.4)	475 (32.2)	76 (59.8)	<0.001

ACEI, angiotensin-converting enzyme inhibitors; ARB, angiotensin-converting enzyme receptor blockers; BNP, brain natriuretic peptide; CABG, coronary artery bypass grafting; CHF, congestive heart failure; DBP, diastolic blood pressure; DM, diabetes mellitus; eGFR, estimated glomerular filtration rate; GRACE, global registry of acute coronary events; HR, heart rate; HDL, high-density lipoprotein; hsTnI, hypersensitive troponin I; HTN, hypertension; LAD, left anterior descending coronary artery; LAD, left atrium diameter; LCX, left coronary circumflex’s artery; LDL, low-density lipoprotein; LM, left main coronary artery; LVEF, left ventricular ejection fraction; MI, myocardial infarction; NLR, neutrophil to lymphocyte ratio; OAC, oral anticoagulants; OHCA, out-of-hospital cardiac arrest; PAD, peripheral arterial disease; PCI, percutaneous coronary intervention; RCA, right coronary artery; SBP, systolic blood pressure; STEMI, ST-elevation myocardial infarction; TC, total cholesterol; TG, triglyceride; WBC, white blood cell.

Overall, NOAF patients had increased values of SHR and NLR than those with SR after AMI regardless of diabetes status. Compared to patients without NOAF, patients with NOAF were older, more frequently females, or had more comorbidities. Moreover, patients with NOAF had higher CHA_2_DS_2_-VASc Score, GRACE score, and Killip classification compared to those with SR. Likewise, AMI cases who developed NOAF had elevated brain natriuretic peptide (BNP) levels, and left atrial dimension but lower eGFR and left ventricular ejection fraction (LVEF). Noteworthy, patients with NOAF experienced prolonged hospital stay compared with the patients in the SR group. At discharge, the use of OAC and diuretics were higher in patients with NOAF than in those without NOAF.

### The incidence of new-onset atrial fibrillation according to quartiles of stress hyperglycemia ratio/neutrophil to lymphocyte ratio

**FIGURE 2:**
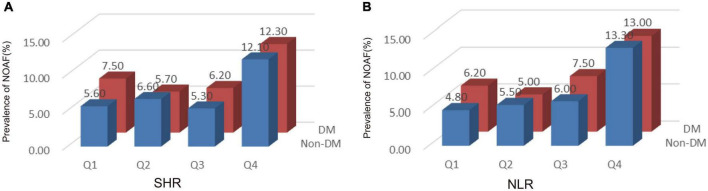
The incidence of new-onset atrial fibrillation (NOAF) in patients with acute myocardial infarction (AMI) according to tertiles of stress hyperglycemia ratio (SHR) **(A)** and neutrophil to lymphocyte ratio (NLR) **(B)**. SHR, stress hyperglycemia ratio; DM, diabetes mellitus; NOAF, new-onset atrial fibrillation.

In our study, a total of 245 AMI patients were newly diagnosed with AF. Figure 2 shows the incidence of NOAF between diabetes and non-diabetes patients with AMI based on the quartiles of SHR/NLR. Regardless of the presence of diabetes, the incidence of NOAF was higher in the Q4 of SHR compared with AMI patients in Q1, Q2, and Q3 of SHR (Figure 2A). Similarly, as shown in Figure 2B, the incidence of NOAF was highest in Q4 of NLR compared with Q1, Q2, and Q3 of NLR regardless of diabetes status.

**FIGURE 3:**
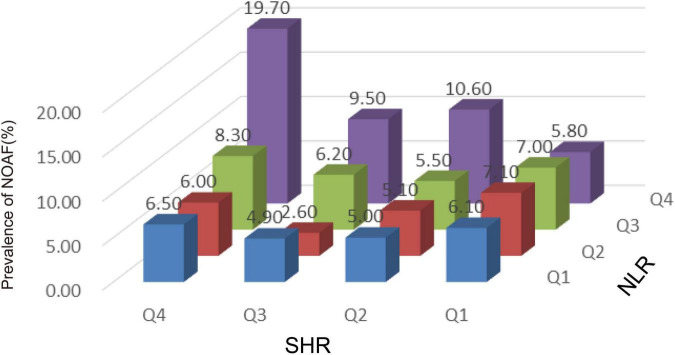
The incidence of new-onset atrial fibrillation (NOAF) in patients with acute myocardial infarction (AMI) based on stress hyperglycemia ratio (SHR) tertiles in patients grouped by neutrophil to lymphocyte ratio (NLR) tertiles. NLR, neutrophil to lymphocyte ratio; SHR, stress hyperglycemia ratio; NOAF, new-onset atrial fibrillation.

The corresponding increase in SHR and NLR was parallel with an increase in NOAF prevalence, suggesting the interaction between SHR and NLR may predict NOAF. As shown in Figure 3, patients in the highest quartile of SHR and NLR had the highest prevalence of NOAF.

### Association between stress hyperglycemia ratio/neutrophil to lymphocyte ratio and the incidence of new-onset atrial fibrillation

The association between SHR and NLR values and the risk of NOAF are given in Table 2 and Supplementary Table 2. We initially treated SHR and NLR as numerical variables. The estimated OR and 95% CI of the SHR and NLR for NOAF were 3.298 (95% CI: 1.415–7.690, *P* = 0.006) and 1.047 (95% CI: 1.013–1.082, *P* = 0.006), respectively. Again, when the values of SHR and NLR were categorized into quartiles, the adjusted OR and 95% CI for NOAF in Q4 of SHR/NLR in relation to their corresponding Q1 was 1.733 (95% CI: 1.130–2.657, *P* = 0.012) and 2.037 (95% CI: 1.341–3.095, *P* = 0.001), respectively.

**TABLE 2:** The prevalence of new-onset atrial fibrillation (NOAF) and the risk estimate for NOAF based on the Q of stress hyperglycemia ratio (SHR).

	Q	No. of NOAF	OR (95% CI)	*P-value*	*P*-trend
NLR	–	–	1.047 (1.013–1.082)	0.006	–
SHR	–	–	3.298 (1.415–7.690)	0.006	–
NLR	Q1 (*n* = 801)	44 (5.5%)	Ref		<0.001
	Q2 (*n* = 799)	42 (5.3%)	0.871 (0.549–1.382)	0.558	
	Q3 (*n* = 796)	54 (6.8%)	0.941 (0.601–1.474)	0.791	
	Q4 (*n* = 798)	105 (13.2%)	2.037 (1.341–3.095)	0.001	
SHR	Q1 (*n* = 794)	52 (6.5%)	Ref		0.007
	Q2 (*n* = 808)	50 (6.2%)	1.100 (0.717–1.689)	0.662	
	Q3 (*n* = 795)	46 (5.8%)	0.887 (0.570–1.381)	0.596	
	Q4 (*n* = 797)	97 (12.2%)	1.733 (1.130–2.657)	0.012	

In patients without DM: SHR-Q1, ≤0.74; SHR-Q2, 0.75–0.81; SHR-Q3, 0.82–0.89; SHR-Q4, >0.89; NLR-Q1, ≤2.46; NLR-Q2, 2.47–3.72; NLR-Q3, 3.73–5.93; NLR-Q4, >5.93; in patients with DM: SHR-Q1, ≤0.72; SHR-Q2, 0.73–0.83; SHR-Q3, 0.84–0.97; SHR-Q4, >0.97; NLR-Q1, ≤2.43; NLR-Q2, 2.44–3.46; NLR-Q3, 3.47–5.64; NLR-Q4, >5.64. Adjusted for age, sex, estimated glomerular filtration rate (eGFR), smoking, congestive heart failure (CHF), KILLIP > 1, heart rate (HR) at admission, left ventricular ejection fraction (LVEF), out-of-hospital cardiac arrest (OHCA), glucose, prior myocardial infarction (MI), prior stroke, left atrium diameter (LAD), log-transformed B-type natriuretic peptide (logBNP), high-density lipoprotein (HDL), low-density lipoprotein (LDL), triglyceride (TG), and percutaneous coronary intervention (PCI) treatment.

### Clinical utility of a nomogram

**FIGURE 4:**
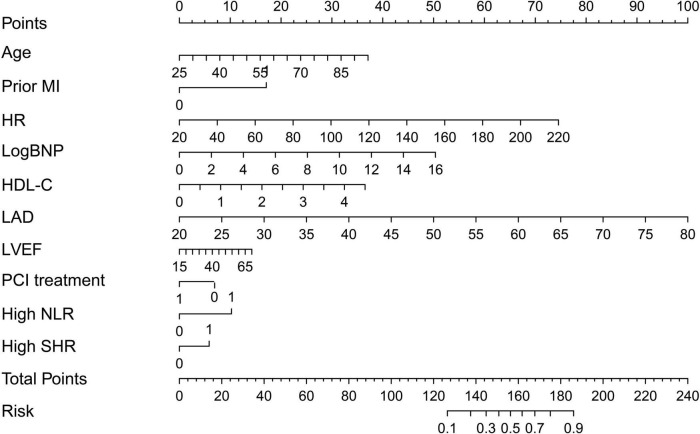
Nomogram to calculate risk score and predict the incidence of new-onset atrial fibrillation (NOAF) in patients with acute myocardial infarction (AMI). MI, myocardial infarction; HR, heart rate; LogBNP, log-transformed B-type natriuretic peptide; HDL, high-density lipoprotein; LAD, left atrium diameter; LVEF, left ventricular ejection fraction; PCI, percutaneous coronary intervention; NLR, neutrophil to lymphocyte ratio; SHR, stress hyperglycemia ratio.

**FIGURE 5:**
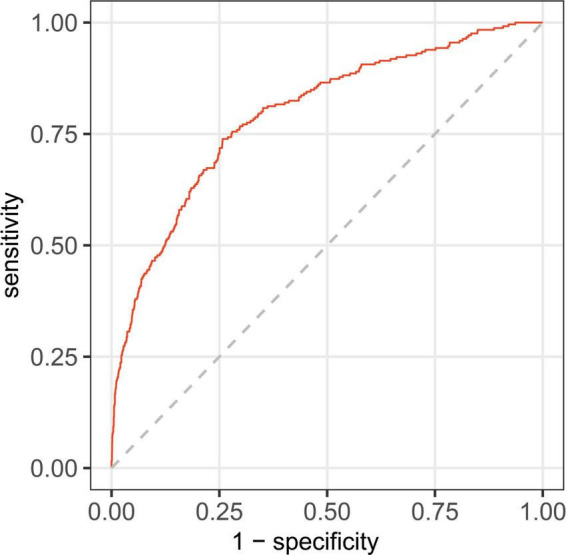
Receiver operating characteristic curve for nomogram in predicting the incidence of new-onset atrial fibrillation (NOAF) in patients with acute myocardial infarction (AMI).

**FIGURE 6:**
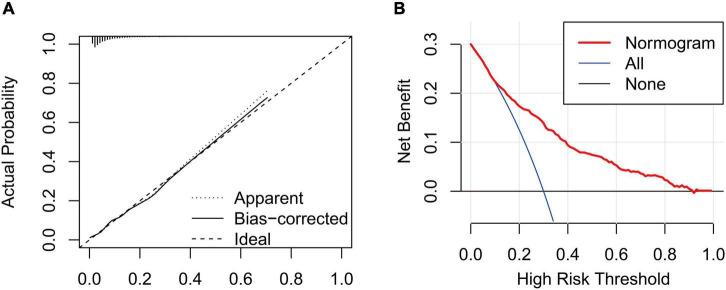
**(A)** Calibration curve for the nomogram to predict the incidence of new-onset atrial fibrillation (NOAF) in patients with acute myocardial infarction (AMI). The *x*-axis represents the predicted incidence of NOAF and the *y*-axis denotes the actual incidence of NOAF. The solid line shows the bias-corrected performance. 1,000 bootstrap repetitions; mean absolute error = 0.005; *n* = 3,194. **(B)** Decision curve analysis for the incidence of NOAF in patients with AMI, implicating the net benefit with respect to the use of the nomogram.

In this study, age, prior MI, HR, log-transformed B-type natriuretic peptide (log BNP), HDL-C, LAD, LVEF, PCI treatment, high NLR, and high SHR were significant factors and were combined to establish a nomogram (Figure 4). As shown in Figure 5, the AUC of the nomogram was 0.795 (95% CI: 0.764–0.825). The calibration plots of the constructed nomogram confirmed that there was an adequate fit between the predicted risk and the observed risk of NOAF with a mean absolute error of 0.005 (Figure 6A). For a threshold probability between 10 and 90%, the nomogram had a higher net benefit for the prediction of NOAF, suggesting the clinical usefulness of the novel nomogram (Figure 6B).

## Discussion

In this study, SHR and NLR were higher in patients with NOAF than in those with non-NOAF after AMI regardless of diabetes status. Also, SHR and NLR were independently associated with NOAF in AMI patients even after adjusting for other traditional risk factors. Importantly, a novel nomogram incorporating high NLR and high SHR was established and showed good prediction performance for NOAF in AMI patients. To the best of our knowledge, we are the first to construct a nomogram incorporating NLR and SHR to predict NOAF in AMI patients.

New-onset atrial fibrillation in patients with AMI is common in clinical practice and is highly associated with the risk of death and prolonged hospital stay (2, 19). The prevalence of NOAF in patients with AMI differs from one study to another (11, 20). In the present study, the prevalence of NOAF was 7.67%, which was consistent with previous studies (20).

The negative impact of SHR and its role in deteriorating the progress of AMI has been described before (12, 21). However, whether the effect of SHR was similar for NOAF in AMI patients with or without DM was not reported precisely. Here, we observed higher SHR values in AMI patients with NOAF than those without NOAF regardless of the diabetic condition. Interestingly, an elevated SHR index was significantly linked with NOAF than ABG or chronic hyperglycemia (surrogated by HbA1c). The mechanisms underlying the association between SHR and NOAF in patients with AMI have not been clearly elucidated, and several reasons may explain their relationship. First, the activation of adrenergic reaction and the flow of catecholamine’s after AMI during stress hyperglycemia may exacerbate inflammation and oxidative stress, which is associated with left atrial dilatation and increased incidence of AF (22, 23). Second, hyperglycemia leads to impaired mitochondrial function which associates with inflammation and oxidative stress (23). Third, the glycation end products formed from hyperglycemia (24) could lead to fibrosis and increased atrial stiffness. An increase in atrial stiffness and left atrial remodeling may be the major factors contributing to the occurrence of AF.

Inflammation plays a role in the repair and remodeling of infarcted heart tissue after the occurrence of AMI (25, 26). Moreover, inflammation participates in the initiation and perpetuation of AF (27). The inflammatory process during AMI involves neutrophils and lymphocytes. Neutrophils represent inflammation whereas lymphocytes represent the inflammatory reaction and the stress phase that exists in the body. In the past, these types of white blood cells (neutrophil and lymphocyte) were found to link with adverse outcomes in AMI cases (28, 29), where elevated neutrophils and depleted lymphocytes were associated with an increased risk of death. Recently, some studies demonstrated that NLR, as the ratio of neutrophil and lymphocyte, which may systematically and accurately reflect the degree of inflammation and stress, was turned out to be superior compared with neutrophil or lymphocyte alone and various leukocyte parameters (10). However, limited studies reported the relationship between NLR and NOAF in patients with AMI. Herein, we found a positive relationship between NLR and NOAF in patients with AMI, even after adjusting for confounding risk factors. Our findings are consistent with previous reports (30–32) highlighting the relationship between inflammation and AF development. Colchicine is an anti-inflammatory drug widely used to treat gout. Recent literature suggested that low-dose colchicine had cardiovascular benefits in patients with acute coronary syndrome through reducing inflammation (33). The effect of anti-inflammatory therapy on NOAF after AMI should also be studied in the future.

Nomograms are of great utility in predicting an individual’s probability of a clinical event using individual factors, and they are common prognostic tools in clinical settings (34, 35). In the present study, we constructed a novel nomogram composed of different risk factors for NOAF. The established nomogram may improve individualized risk stratification and tailor the individual therapy strategy for the primary prevention of NOAF in AMI patients. The satisfactory performance of this model was reflected by an AUC of more than 0.79. In addition, the calibration curve shows the prediction of NOAF in patients with AMI was similar to the actual NOAF in patients with AMI. Furthermore, decision curve analysis indicated that the nomogram would gain more net benefits with threshold probabilities of 0.1–0.9.

## Limitation

First of all, we could not exclude the possibility of selection bias given that part of the subjects were excluded due to missing data. Also, the number of patients who developed NOAF in this study is limited, further prospective, multicenter, large-sample studies are highly desirable. Second, we recorded the existence of NOAF only by ECG, which may underestimate the incidence of AF in our study. It is also possible that some patients with pre-existing asymptomatic AF have been erroneously regarded to have NOAF. Thirdly, very few patients had intensive ECG Holter monitoring after discharge, as a result, we have only data regarding NOAF during hospitalization period in patients with AMI. Future studies with long-term follow up are required. Finally, the nomogram has not been verified in the external validation queue, thus, external validations are needed to verify our findings in the future.

## Conclusion

SHR and NLR are positively linked with the incidence of NOAF in patients with AMI. Furthermore, the novel nomogram might be helpful for risk stratification and tailoring the individual therapy strategy for the primary prevention of NOAF in patients with AMI.

## Data availability statement

The raw data supporting the conclusions of this article will be made available by the authors, without undue reservation.

## Ethics statement

The studies involving human participants were reviewed and approved by the Ethics Committee of the First Affiliated Hospital of Dalian Medical University. Written informed consent for participation was not required for this study in accordance with the national legislation and the institutional requirements.

## Author contributions

YL and LZ designed the study. LP and ZL were in charge of the data analysis. LP drafted the manuscript. LP, ZL, CL, XD, and FL conducted the data collection. TH, YX, and XY did the critical revision of the manuscript. All authors have read and approved the final manuscript.

## Footnotes

1
http://www.r-project.org/
