# Supplementary material for: Stress hyperglycemia ratio and neutrophil to lymphocyte ratio are reliable predictors of new-onset atrial fibrillation in patients with acute myocardial infarction
Source: Front Cardiovasc Med. 2022 Nov 9;9:1051078. doi: 10.3389/fcvm.2022.1051078 (PMC9681791; doi:10.3389/fcvm.2022.1051078)
Supplement: Supplementary file 1 [file Data_Sheet_1.docx]

**Supplementary materials**

**Table S1.Baseline Characteristics**

| Variable | Overall | AF/AFL rhythm at admission | Sinus rhythm at admission | | *P*-Value |
| --- | --- | --- | --- | --- | --- |
|  | (n=245) | (n=72) | | (n=173) |  |
| Age, years | 69.6(9.9) | 68.9(10.3) | | 69.9(9.7) | 0.488 |
| Male, n (%) | 160(65.3) | 45(62.5) | | 115(66.5) | 0.552 |
| Smoking, n (%) | 82(33.5) | 25(34.7) | | 57(32.9) | 0.789 |
| Drinking, n (%) | 43(17.6) | 18(25.0) | | 25(14.5) | **0.048** |
| **Medical history** | |  | |  |  |
| HTN, n (%) | 158(64.5) | 45(62.5) | | 113(65.3) | 0.675 |
| DM, n (%) | 127(51.8) | 36(50.0) | | 91(52.6) | 0.710 |
| Prior MI, n (%) | 7(2.9) | 1(1.4) | | 6(3.5) | 0.639 |
| Prior PCI, n (%) | 24(9.8) | 11(15.3) | | 13(7.5) | 0.063 |
| Previous stroke, n (%) | 27(11.0) | 9(12.5) | | 18(10.4) | 0.633 |
| PAD, n (%) | 31(12.7) | 10(13.9) | | 21(12.1) | 0.707 |
| CHF, n (%) | 40(16.3) | 14(19.4) | | 26(15.0) | 0.394 |
| **Initial presentation** | |  | |  |  |
| SBP,mmHg | 131.8(25.8) | 132.2(25.7) | | 131.7(25.9) | 0.886 |
| DBP,mmHg | 78.9(14.5) | 80.6(15.1) | | 78.2(14.3) | 0.243 |
| HR at admission, b.p.m. | 84.4(23.4) | 91.0(25.4) | | 81.7(22.0) | **0.004** |
| KILLIP>1, n (%) | 83(33.9) | 28(38.9) | | 55(31.8) | 0.285 |
| OHCA, n (%) | 7(2.9) | 2(2.8) | | 5(2.9) | 1.000 |
| STEMI, n (%) | 126(51.4) | 34(47.2) | | 92(53.2) | 0.395 |
| Anterior wall, n (%) | 49(38.9) | 14(41.2) | | 35(38.0) | 0.749 |
| Inferior wall, n (%) | 44(34.9) | 12(35.3) | | 32(34.8) | 0.720 |
| Others, n (%) | 19(15.1) | 7(20.6) | | 12(13.0) | 0.293 |
| CHA_2_DS_2_-VASc Score | 3(2-4) | 3(1-5) | | 3(2-4) | 0.892 |
| GRACE Score | 153(130-176) | 153(130-179) | | 153(129-176) | 0.957 |
| **Culprit lesion** |  |  | |  |  |
| LM, n (%) | 8(3.3) | 2(2.8) | | 6(3.5) | 1.000 |
| LAD, n (%) | 111(45.3) | 33(45.8) | | 78(45.1) | 0.915 |
| LCX, n (%) | 44(18.0) | 8(11.1) | | 36(20.8) | 0.072 |
| RCA, n (%) | 77(31.4) | 26(36.1) | | 51(29.5) | 0.308 |

**Table S1 (continued)**

| Variable | Overall | AF/AFL rhythm at admission | | Sinus rhythm at admission | | *P*-Value |
| --- | --- | --- | --- | --- | --- | --- |
|  | (n=245) | | (n=72) | | (n=173) |  |
| **Laboratory data and ECG parameters** | | | | |  |  |
| eGFR,ml/(min ▪1.73 m^2^) | 83.0(25.0) | | 84.1(22.6) | | 82.6(26.0) | 0.673 |
| BNP,pg/mL | 303(137-645) | | 377(162-744) | | 280(131-505) | 0.137 |
| hsTnI,pg/mL | 19.1(1.6-80.6) | | 22.0(1.6-102.0) | | 18.0(1.6-76.0) | 0.172 |
| Glucose, mmol/L | 6.6(5.2-9.4) | | 6.9(5.4-10.3) | | 6.3(5.2-9.4) | 0.591 |
| HbA1c(%) | 6.3(5.7-8.2) | | 6.5(5.7-8.4) | | 6.3(5.7-8.0) | 0.818 |
| SHR | 0.9(0.7-1.0) | | 0.9(0.7-1.0) | | 0.9(0.7-1.0) | 0.900 |
| WBC,*10^9/L | 9.0(7.1-11.2) | | 8.3(7.2-10.7) | | 9.1(7.1-11.5) | 0.204 |
| Neutrophil,*10^9/L | 6.7(4.9-9.0) | | 6.1(4.9-8.7) | | 6.9(4.9-9.1) | 0.157 |
| Lymphocyte,*10^9/L | 1.4(1.0-1.9) | | 1.4(1.1-2.0) | | 1.4(1.0-1.8) | 0.322 |
| NLR | 4.7(2.9-7.7) | | 4.6(2.5-6.9) | | 4.9(3.1-8.4) | 0.128 |
| Hemoglobin,g/L | 136(124-145) | | 137(124-146) | | 136(124-145) | 0.926 |
| Platelet,*10^9/L | 202(163-236) | | 205(157-240) | | 200(164-234) | 0.903 |
| TC, mmol/L | 4.6(3.9-5.4) | | 4.5(3.9-5.3) | | 4.6(4.0-5.5) | 0.399 |
| TG, mmol/L | 1.3(1.0-1.9) | | 1.2(1.0-2.0) | | 1.3(1.0-1.9) | 0.980 |
| HDL, mmol/L | 1.1(0.9-1.2) | | 1.0(0.9-1.2) | | 1.1(0.9-1.3) | 0.968 |
| LDL, mmol/L | 2.6(2.1-3.1) | | 2.6(2.1-3.1) | | 2.6(2.1-3.2) | 0.635 |
| **Echocardiographic parameters** | | |  | |  |  |
| LAD, mm | 39.8(5.6) | | 41.9(7.1) | | 38.9(4.6) | **0.001** |
| LVEF, % | 48.8(9.3) | | 48.1(9.2) | | 49.1(9.3) | 0.462 |
| **Initial treatment** | | |  | |  |  |
| PCI, n (%) | 189(77.1) | | 56(77.8) | | 133(76.9) | 0.879 |
| CABG, n (%) | 2(0.8) | | 0(0.0) | | 2(1.2) | 1.000 |
| Thrombolysis, n (%) | 3(1.2) | | 0(0.0) | | 3(1.7) | 0.558 |
| Length of hospitalization, day | 7(6-9) | | 6(6-8) | | 7(6-10) | **0.003** |
| In-hospital death, n (%) | 4(1.6) | | 1(1.4) | | 3(1.7) | 1.000 |
| **Medication at discharge** | | |  | |  |  |
| ACEI/ARB, n (%) | 164(66.9) | | 44(61.1) | | 120(69.4) | 0.211 |
| βblocker, n (%) | 218(89.0) | | 64(88.9) | | 154(89.0) | 0.977 |
| Statins, n (%) | 245(100.0) | | 72(100.0) | | 173(100.0) | 1.000 |
| OAC, n (%) | 47(19.2) | | 26(36.1) | | 21(12.1) | **<0.001** |
| Aspirin, n (%) | 220(89.8) | | 51(70.8) | | 169(97.7) | **<0.001** |
| P2Y_12_ receptor inhibitor, n (%) | 240(98.0) | | 67(93.1) | | 173(100.0) | **0.003** |
| Diuretic, n (%) | 133(54.3) | | 32(44.4) | | 101(58.4) | **0.046** |

Abbreviations: ACEI=angiotensin-Converting Enzyme Inhibitors; ARB=angiotensin-converting enzyme receptor blockers; BNP=brain natriuretic peptide; CABG=coronary artery bypass grafting; CHF=Congestive heart failure; DBP=diastolic blood pressure; DM=Diabetes Mellitus; eGFR=estimated glomerular filtration rate; GRACE: global registry of acute coronary events; HR=heart rate; HDL=high-density lipoprotein; hsTnI=hypersensitive troponin I; HTN=Hypertension; LAD=left anterior descending coronary artery; LAD=left atrium diameter; LCX=left coronary circumflexus artery; LDL= low-density lipoprotein; LM=left main coronary artery; LVEF= left ventricular ejection fraction; MI=myocardial infarction; NLR= Neutrophil to lymphocyte ratio; OAC=oral anticoagulants; OHCA:out-of-hospital cardiac arrest; PAD=peripheral arterial disease; PCI=percutaneous coronary intervention; RCA=right coronary artery; SBP=systolic blood pressure; STEMI=ST-elevation myocardial infarction; TC=Total Cholesterol; TG=triglyceride; WBC=White blood cell.

**Table S2.Univariate and Multivariate Logistic Regression Analysis of NOAF**

| Variable | Univariate analysis | | Multivariate analysis | |
| --- | --- | --- | --- | --- |
|  | OR(95%CI) | P value | OR(95%CI) | P value |
| Age, years | 1.059(1.045-1.072) | <0.001 | 1.034(1.018-1.050) | **<0.001** |
| Male sex | 0.552(0.419-0.728) | <0.001 | 0.871(0.598-1.268) | 0.470 |
| Smoking | 0.585(0.444-0.770) | <0.001 | 0.901(0.637-1.272) | 0.552 |
| Drinking | 0.762(0.542-1.071) | 0.118 |  |  |
| **Medical history** |  |  |  |  |
| HTN | 1.253(0.955-1.644) | 0.104 |  |  |
| DM | 1.078(0.831-1.400) | 0.570 |  |  |
| Prior MI | 3.742(1.589-8.810) | 0.003 | 3.754(1.495-9.430) | **0.005** |
| Prior PCI | 1.172(0.755-1.822) | 0.479 |  |  |
| Previous stroke | 1.615(1.057-2.468) | 0.027 | 0.825(0.506-1.345) | 0.440 |
| PAD | 1.359(0.915-2.020) | 0.129 |  |  |
| CHF | 2.558(1.773-3.692) | <0.001 | 0.919(0.585-1.443) | 0.712 |
| **Initial presentation** | |  |  |  |
| SBP,mmHg | 0.998(0.993-1.004) | 0.536 |  |  |
| DBP,mmHg | 1.000(0.990-1.010) | 0.922 |  |  |
| HR at admission, b.p.m. | 1.034(1.026-1.042) | <0.001 | 1.028(1.020-1.036) | **<0.001** |
| KILLIP>1 | 2.875(2.166-3.816) | <0.001 | 1.162(0.818-1.650) | 0.403 |
| OHCA | 2.449(1.076-5.572) | 0.033 | 2.352(0.915-6.048) | 0.076 |
| STEMI | 1.162(0.895-1.508) | 0.259 |  |  |
| **Culprit lesion** |  |  |  |  |
| LM | 1.572(0.744-3.321) | 0.236 |  |  |
| LAD | 1.134(0.872-1.473) | 0.348 |  |  |
| LCX | 0.955(0.680-1.340) | 0.789 |  |  |
| RCA | 0.838(0.633-1.108) | 0.215 |  |  |
| **laboratory data** |  |  |  |  |
| eGFR, ml/(min ▪1.73 m^2^) | 0.985(0.979-0.990) | <0.001 | 0.998(0.992-1.004) | 0.538 |
| LogBNP | 1.513(1.400-1.636) | <0.001 | 1.242(1.120-1.378) | **<0.001** |
| hsTnI,pg/mL | 1.000(1.000-1.001) | 0.402 |  |  |
| Glucose(mmol/L) | 1.073(1.036-1.111) | <0.001 | 0.993(0.950-1.038) | 0.755 |
| HbA1c(%) | 1.012(0.943-1.087) | 0.738 |  |  |
| High SHR | 2.106(1.608-2.758) | <0.001 | 1.574(1.124-2.203) | **0.008** |
| High NLR | 2.442(1.870-3.189) | <0.001 | 2.054(1.508-2.796) | **<0.001** |
| TC, mmol/L | 0.898(0.799-1.008) | 0.069 |  |  |
| TG, mmol/L | 0.696(0.591-0.820) | <0.001 | 0.945(0.824-1.085) | 0.422 |
| HDL, mmol/L | 2.107(1.348-3.292) | 0.001 | 1.933(1.147-3.257) | **0.013** |
| LDL, mmol/L | 0.828(0.704-0.975) | 0.023 | 0.862(0.716-1.037) | 0.116 |
| **Echocardiographic parameters** | |  |  |  |
| LAD, mm | 1.156(1.120-1.192) | <0.001 | 1.135(1.095-1.177) | **<0.001** |
| LVEF, % | 0.959(0.947-0.972) | <0.001 | 1.021(1.002-1.040) | **0.028** |
| **Initial treatment** |  |  |  |  |
| PCI | 0.567(0.413-0.777) | <0.001 | 0.626(0.441-0.890) | **0.009** |
| CABG | 1.340(0.309-5.810) | 0.696 |  |  |
| Thrombolysis | 1.649(0.490-5.550) | 0.419 |  |  |

Abbreviations: BNP=brain natriuretic peptide; CABG=coronary artery bypass grafting; CHF=Congestive heart failure; DBP=diastolic blood pressure; DM=Diabetes Mellitus; eGFR=estimated glomerular filtration rate; HR=heart rate; HDL=high-density lipoprotein; hsTnI=hypersensitive troponin I; HTN=Hypertension; LAD=left anterior descending coronary artery; LAD=left atrium diameter; LCX=left coronary circumflexus artery; LDL= low-density lipoprotein; LM=left main coronary artery; LVEF= left ventricular ejection fraction; MI=myocardial infarction; NLR= Neutrophil to lymphocyte ratio; OHCA:out-of-hospital cardiac arrest; PAD=peripheral arterial disease; PCI=percutaneous coronary intervention; RCA=right coronary artery; SBP=systolic blood pressure; STEMI=ST-elevation myocardial infarction; TC=Total Cholesterol; TG=triglyceride;
